# Bispecific antibodies for the treatment of relapsed/refractory multiple myeloma: updates and future perspectives

**DOI:** 10.3389/fonc.2024.1394048

**Published:** 2024-04-10

**Authors:** Ricardo D. Parrondo, Sikander Ailawadhi, Claudio Cerchione

**Affiliations:** ^1^ Division of Hematology-Oncology and Blood and Marrow Transplantation Program, and Cellular Therapies, Mayo Clinic Comprehensive Cancer Center, Jacksonville, FL, United States; ^2^ Hematology Unit, Istituto Scientifico Romagnolo per lo Studio e la Cura dei Tumori (IRST) IRCCS, Meldola, Italy

**Keywords:** multiple myeloma, bispecific antibodies, immunotherapy, T-cell engagers, T-cell redirecting therapy

## Abstract

Patients with relapsed/refractory multiple myeloma (RRMM) that are refractory to the five most active anti-MM drugs, so-called penta-refractory MM, have historically had dismal outcomes with subsequent therapies. Progressive immune dysfunction, particularly of the T-cell repertoire, is implicated in the development of disease progression and refractory disease. However, the advent of novel immunotherapies such as bispecific antibodies are rapidly changing the treatment landscape and improving the survival outcomes of patients with RRMM. Bispecific antibodies are antibodies that are engineered to simultaneously engage cytotoxic immune effector cells (T cells or NK cells) and malignant plasma cells via binding to immune effector cell antigens and extracellular plasma cell antigens leading to immune effector cell activation and malignant plasma cell destruction. Currently, bispecific antibodies that bind CD3 on T cells and plasma cell epitopes such as B-cell maturation antigen (BCMA), G-protein coupled receptor family C group 5 member D (GPRC5d), and Fc receptor homologue 5 (FcRH5) are the most advanced in clinical development and are showing unprecedented response rates in patients with RRMM, including patients with penta-refractory disease. In this review article, we explore the available clinical data of bispecific antibodies in RRMM and summarize the efficacy, safety, toxicity, clinical outcomes, mechanisms of resistance, and future directions of these therapies in patients with RRMM.

## Introduction

Due to the advent of novel agents such as proteasome inhibitors (PI), immunomodulatory agents (IMiD), and anti-CD38 monoclonal antibodies (MoAb), the survival of patients with multiple myeloma (MM) has improved and is expected to continue to improve (1, 2). Nonetheless, relapse is inevitable with subsequent remissions being shorter due to acquired drug resistance and development of refractory disease (3). Patients that are triple-class refractory (refractory to a PI, IMiD, and anti-CD38 MoAb) have a dismal prognosis with a reported overall response rate (ORR) of 31%, a median progression free survival (PFS) of 3.4 months and a median overall survival (OS) of 9.3 months with the subsequent treatment regimens following anti-CD38 MoAb failure (4). Additionally, patients with penta-refractory myeloma (myeloma refractory to lenalidomide, pomalidomide, bortezomib, carfilzomib and an anti-CD38 MoAb) have an even more grim prognosis with a median OS of about 6 months with subsequent therapies (5–7).

**Figure 1:**
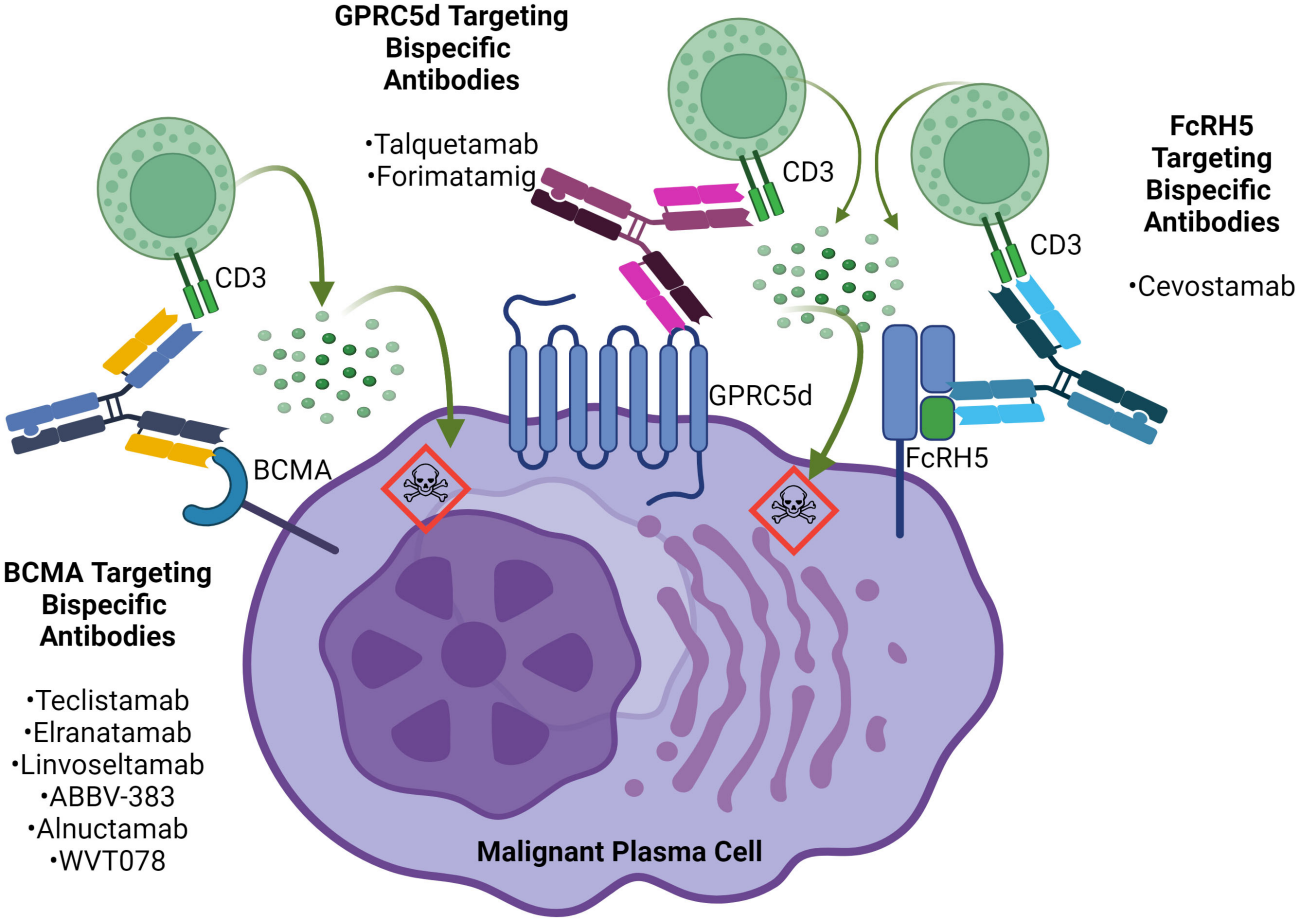
BCMA, GPRC5d and FcRH5-targeting bispecific antibodies in multiple myeloma.

Ineffective T-cell immunity has been associated with the development of RRMM and disease progression due to mechanisms such as T-cell anergy, exhaustion and senescence (8). However, studies evaluating the endogenous T-cells of patients with RRMM that have undergone ex-vivo stimulation reveal that once stimulated, those T-cells can mount anti-myeloma cytotoxic activity (9, 10). Bispecific antibodies are therapeutic agents designed to simultaneously engage endogenous T cells and malignant cells via binding to a T-cell epitope (usually CD3) and an extracellular tumor antigen which in turn stimulates cytotoxic T cell activity and the release of cytotoxic granules leading to tumor cell death (11). Bispecific antibodies targeting plasma-cell antigens such as B-cell maturation antigen (BCMA), G-protein coupled receptor family C group 5 member d (GPRC5d), and Fc receptor-homolog 5 (FcRH5) are very advanced in clinical development with teclistamab, elranatamab (BCMA-targeting bispecific antibodies) and talquetamab (GPRC5d) already FDA approved for RRMM after ≥4 or more lines of therapy and other anti-BCMA and GPRC5d bispecific antibodies and cevostamab (FcRH5 x CD3 bispecific antibody) in clinical development (**Figure 1**). In this review article, we summarize all the available clinical data evaluating the use of bispecific antibodies for RRMM as well as ongoing clinical trials, bispecific antibodies in development, mechanisms of resistance and future directions with bispecific antibodies in patients with RRMM. A systematic literature review was performed in PubMed and across all abstracts from relevant congresses, ASCO, EHA, and ASH, from January 1, 2019 until February 1, 2024 to identify relevant information about bispecific antibodies in patients with multiple myeloma, using the search terms of “multiple myeloma”, “bispecific T-cell engagers”, “bispecific antibodies,” “trispecific antibodies,” and “NK cell engagers”. We also used the search terms “teclistamab,” “elranatamab,” “linvoseltamab,” “ABBV-383,” “alnuctamab,” “WVT078,” “talquetamab,” “forimtamig,” “cevostamab,” “ISB 1342,” and “ISB 1442”. Primary articles that were published in English were assessed for relevancy, to ensure inclusion of all papers and abstracts with clinical data with bispecific antibodies. For clinical trials with multiple data cutoffs, the most recent data were used.

## BCMA-targeting bispecific antibodies

BMCA is a member of the tumor necrosis factor receptor superfamily and plays a crucial role in the survival of long-lived bone marrow plasma cells (12, 13). Additionally, the overexpression of serum BCMA correlates with disease progression and shorter PFS and OS in patients with MM making BCMA an attractive therapeutic target (14, 15).

### Teclistamab

Teclistamab is a bispecific antibody that targets BCMA expressed on the surface of myeloma cells and CD3 on the surface of T cells. In the multicohort phase I/II MajesTEC-1 study (NCT03145181 and NCT04557098), 157 patients who had received a median of 6 prior lines of therapy (82% triple-class refractory and 39% penta-drug refractory) received at least one dose of teclistamab. The recommended phase 2 dose (RP2D) for teclistamab was 1500 µg/kg (after 60 µg/kg and 300 µg/kg step-up doses) and at the RP2D, the ORR was 65% (n=26/40) and the median duration of response (DOR) was not reached after 7.1 months of follow-up. Cytokine release syndrome (CRS) occurred in 70% (n=28/40) of patients in the RP2D cohort but was all grade 1 or 2. One patient developed grade 1 neurotoxicity in the RP2D cohort and 40%, 28%, and 20% developed ≥grade 3 neutropenia, anemia, and thrombocytopenia, respectively. Infections were reported in 18 (45%) of the 40 patients treated in the RP2D cohort of which 23% (n=9) had ≥grade 3 infection (16). In the phase II portion of the MajesTEC-1 study, 165 patients (including 40 patients at the RP2D from phase 1) received teclistamab at the RP2D. Patients had received a median of 5 prior lines of therapy, 100% of patients were triple-class refractory and 70.3% were penta-drug refractory. After a median follow-up of 14.1 months, the ORR was 63% (n=104/165), the minimal residual disease negativity (MRD) rate to 10^-5^ was 26.7% (n=44/165), the median DOR was 18.4 months, the median PFS was 11.3 months and the median OS was 18.3 months. Any grade neutropenia, anemia and thrombocytopenia occurred in 70.9% (64.2% ≥grade 3), 51.1% (37% ≥grade 3) and 40% (21.1% ≥grade 3) of patients, respectively. Infections occurred in 76.4% (n=126) patients with 44.8% (n=74) having ≥grade 3 infections. CRS occurred in 72.1% (n-119) of patients, most of which occurred after step-up and cycle 1 doses and almost all CRS events were grade 1 or 2. Immune effector cell–associated neurotoxicity syndrome (ICANS) events were reported in 14.5% of patients (n=24) and most events were grade 1 or 2, except for one grade 4 seizure event (17). The results of the MajesTEC-1 study led to the FDA approval of teclistamab for patients with RRMM after ≥4 prior lines of therapy on October 25, 2022. In one of the MajesTEC-1 study cohorts, patients who had received the RP2D (1.5mg/kg weekly) had the option to switch from 1.5mg/kg weekly dosing to every 2 week (Q2W) dosing if they achieved a confirmed partial response or better after ≥4 cycles of treatment (phase 1) or a confirmed complete response (CR) or better for ≥6 months (phase 2). Of the 165 patients who received teclistamab at the RP2D, 104 were responders and 60 patients switched to Q2W dosing. At a median of 11.1-months of follow-up since switching to Q2W dosing, the median duration of response from the date of switch was 20.5 months with 40 of 60 patients still in response with ongoing treatment (18). Based on these results, the FDA approved Q2W dosing of teclistamab for patients who have achieved and maintained a complete response or better for a minimum of 6 months on 1.5mg/kg weekly on February 20, 2024. Other studies evaluating teclistamab in combination with other anti-myeloma therapies in both newly diagnosed MM and RRMM are underway (**Table 1**).

**Table 1:** Ongoing clinical trials evaluating teclistamab in multiple myeloma.

Clinical Trials.Gov Identifier	Clinical Trial Name	Phase	Estimated Enrollment	Population	Treatment	Primary Endpoint
NCT04722146	MajesTEC-2	1b	140	NDMM and RRMM	Teclistamab in combination with: • Daratumumab +Pomalidomide • Daratumumab + Lenalidomide • Daratumumab + Bortezomib + Lenalidomide • Lenalidomide • Nirogacestat	Safety and Tolerability
NCT05083169	MajesTEC-3	III	587	RRMM	Teclistamab + Daratumumab Vs. Daratumumab + Pomalidomide +Dexamethasone Vs. Daratumumab + Bortezomib +Dexamethasone	PFS
NCT05243797	MajesTEC-4	III	1000	NDMM	Teclistamab + Lenalidomide vs. Lenalidomide as maintenance therapy for NDMM patients in the post ASCT setting	PFS
NCT05695508	MajesTEC-5	II	70	NDMM	Teclistamab + Daratumumab + Lenalidomide+ Dexamethasone Vs. Teclistamab + Daratumumab + Bortezomib+ Lenalidomide+ Dexamethasone	Safety and Tolerability
NCT05552222	MajesTEC-7	III	1590	NDMM (Transplant Ineligible)	Teclistamab + Daratumumab + Lenalidomide Vs. Talquetamab + Daratumumab + Lenalidomide Vs. Daratumumab + Lenalidomide + Dexamethasone	PFS
NCT05572515	MajesTEC-9	III	590	RRMM	Teclistamab Vs. Bortezomib + Pomalidomide + Dexamethasone Or Carfilzomib + Dexamethasone	PFS

ASCT, Autologous Stem Cell Transplant; NDMM, Newly Diagnosed Multiple Myeloma; RRMM, Relapsed Refractory Multiple Myeloma; PFS, Progression Free Survival.

The phase 1b MajesTEC-2 study (NCT04722146) is a multicohort study evaluating teclistamab in combination with other anti-myeloma agents. The cohort evaluating teclistamab in combination with the gamma-secretase inhibitor nirogacestat has reported preliminary results. As of Dec 16, 2022, 28 patients who had received a median of 4 prior lines of therapy received teclistamab + nirogacestat at 3 different doses levels; 1) teclistamab 720 μg/kg weekly with concurrent nirogacestat (100 mg twice daily starting with the first dose of teclistamab [n=8]) or 2) teclistamab 720 μg/kg weekly + once daily delayed low-dose nirogacestat (100 mg daily starting after teclistamab step-up dosing [n=7]) or 3) teclistamab 1500 μg/kg weekly + daily delayed low dose nirogacestat (n=13). There were three dose-limiting toxicities (grade 3 gastrointestinal bleeding, grade 3 diarrhea, and grade 3 ICANS) with teclistamab 720 μg/kg weekly with concurrent nirogacestat whereas the cohorts with delayed low dose nirogacestat did not develop any dose-limiting toxicities or grade 3 CRS or ICANS. The most frequent treatment-related adverse events for all doses occurring in >20% of patients were neutropenia (82.1%), CRS (75%), diarrhea (64.3%), injection-site erythema (53.6%), decreased appetite (50%), fatigue (42.9%), and anemia (35.1%). The ORR was 71.4% (n=5/8) for teclistamab 720 μg/kg weekly with concurrent nirogacestat, 57.1% (n=4/7) for teclistamab 720 μg/kg weekly + once daily delayed low-dose nirogacestat, and 92.3% (n=12/13) for teclistamab 1500 μg/kg weekly + daily delayed low dose nirogacestat (19). The cohort evaluating teclistamab in combination with subcutaneous daratumumab and lenalidomide has also reported preliminary results. Thirty-two patients received teclistamab-daratumumab-lenalidomide at two different dose levels: 1) teclistamab at 0.72 mg/kg (n=13) or 2) teclistamab at 1.5 mg/kg (n=19). The median age was 62 years and patients had received a median of 2 prior lines of therapy with 31.3% of patients being anti-CD38 monoclonal antibody exposed. After a median follow up of 5.78 months, the most frequent adverse event was CRS (81.3% [n=26]) and all CRS events were grade 1/2, with 95% of the CRS events occurring during cycle 1 of treatment. No ICANS events were reported. Other frequent adverse events occurring in ≥25.0% of patients across both dose level were neutropenia (75.0% [n=24]), fatigue (43.8% [n=14]), diarrhea (37.5% [n=12]), insomnia (31.3% [n=10]), cough (28.1% [n=9]), hypophosphatemia (25.0% [n=8]), and pyrexia (25% [n=8]). The ORR was 100% (n=13/13) at 0.72 mg/kg and 81.2% (n=13/16) at 1.5mg/kg (20).

There are several studies underway as well as real-world data, evaluating modifications to teclistamab dosing as well as teclistamab administration in select myeloma patient populations. LimiTec (NCT05932680) is a phase II, single-arm, non-inferiority study with an estimated enrollment of 75 patients, exploring whether limited-duration teclistamab therapy is non-inferior to continuously administered therapy in patients who have received 6 to 9 months of teclistamab and have achieved ≥ very good partial response (VGPR) (21). Accrual for this study started in July 2023. The package insert of teclistamab states that due to risk of CRS and ICANS, patients should be hospitalized for 48 hours after administration of all step-up doses of teclistamab (22). Optec is a phase II study (NCT05972135) that will evaluate CRS incidence and severity after prophylactic tocilizumab in patients receiving teclistamab in the outpatient setting with a target enrollment of 50 patients (23). In the MajesTEC-1 study, patients who received one dose of IV tocilizumab before the first teclistamab step-up dose (n=23) had a lower incidence of all grade CRS compared with the overall study population; 26% vs. 72%, respectively, hence further evaluation of CRS prophylaxis in patients receiving teclistamab is warranted (17, 24). Emerging real-world data is already showing the feasibility of outpatient administration of teclistamab and the benefit of CRS prophylaxis with tocilizumab. At the tri-site Mayo Clinic Comprehensive Cancer Center (MCCC), teclistamab step-up dosing is administered in an outpatient setting with patients given a remote monitoring kit to regularly measure vital signs and stay connected with a command center for signs and symptoms of CRS and ICANS throughout the step-up dosing period. In a retrospective study of thirty-seven patients from the MCCC who completed outpatient step up dosing of teclistamab using a remote monitoring kit, 32% (n=12) developed CRS. The highest CRS grade was grade 1 for 10 patients; 1 patient had a grade 2 CRS, and 1 patient had a grade 4 CRS. All 12 patients who developed CRS during the step-up dosing period were admitted to the hospital for treatment, with a total of 19 admissions across all patients and a median length of stay of 1.7 days per admission (25). In a single center retrospective study of 31 patients who received a single dose of prophylactic tocilizumab prior to initiation of teclistamab, a low rate of CRS (13%) and ICANS (10%) was observed. CRS was limited to grade 1 and there was 1 episode of grade 2 neurotoxicity (26).

Another retrospective study evaluated the safety and feasibility of administering teclistamab to patients on hemodialysis, a patient population that was excluded from the MajesTEC-1 trial. Thirteen patients from French hospitals with end-stage renal disease on hemodialysis, who had received a median of 4 prior lines of therapy, received teclistamab with standard step-up dosing (1-2 days after hemodialysis). Half of the patients developed CRS of grade 1 or 2, treated with tocilizumab and no ICANS was reported. With a median follow-up of 4 months, no disease progression or deaths were reported (27). Further evaluation of teclistamab in patients on hemodialysis is warranted.

Teclistamab has also proven to be efficacious in RRMM patients who have received prior BCMA-targeted therapy. In cohort C of the MajecTEC-1 trial (which allowed prior BCMA-targeted therapy), 38 patients who had received a median of 6 prior lines of therapy received teclistamab and 25 (66%) of those patients were refractory to an anti-BCMA -directed therapy. Sixteen patients (64%) received prior BCMA-targeting antibody drug conjugate, and 11 (44%) received prior BCMA-targeting CAR-T. At a median follow-up of 6.9 months, the ORR was 40% and ≥CR was observed in 5 patients (20%). In the BCMA-directed antibody drug conjugate-exposed and anti-BCMA CAR-T-exposed patients, the ORR was 38% and 45%, respectively (28). A retrospective multicenter study evaluated 105 patients with RRMM who received teclistamab, 65% were penta-drug refractory, 83% would have been considered ineligible for the MajesTEC-1 trial, and 53% received prior BCMA-targeting therapy. In this real-world analysis, teclistamab led to an ORR of 66%, including a ≥CR rate of 29% in the entire cohort whereas patients who received prior-BCMA targeted therapy (75% prior BCMA-targeted CAR-T and 41% prior belantamab mafodotin) achieved an ORR of 59% (29). In another retrospective analysis of 123 patients (65% penta-drug refractory) with RRMM who had received teclistamab across 18 different German centers, 37.4% (n=45) had received prior BCMA-directed therapy; 17.1% (n=21) had received prior idecabtagene vicleucel (ide-cel) and 18.7% (n=23) had received prior belantamab mafodotin. The ORR for the entire cohort was 59.3% with 22.0% achieving CR. With a median follow-up of 5.5 months, the median PFS was 8.7 months. The ORR for patients with prior BCMA-targeted therapy was lower at 54.8% compared to anti-BCMA naive patients with an ORR of 64.5%. This difference was exclusively attributable to patients pretreated with ide-cel (n = 21) who had an ORR of only 33.3%. However, the ORR for patients with previously treated with belantamab mafodotin (73.9%) was comparable to the ORR in anti-BCMA naive patients (64.5%). Patients who had received prior ide-cel had a significantly lower median PFS of 1.8 months (30). Another retrospective real-world analysis which included comprehensive pre-treatment immune profiling, reported on the efficacy of teclistamab in 52 patients with RRMM who received commercial teclistamab. Fifty-two percent (n=27) of patients had prior exposure to anti-BCMA therapies, including belantamab mafodotin in 31% (n=16/52), anti-BCMA CAR T cell therapies in 37% (n=19/52), and anti-BCMA bispecific antibodies in 4% (n=2/52). The ORR was 64% (n=30/47) in the 47 response evaluable patients in the entire cohort. Amongst the 26 response evaluable patients who had received prior anti-BCMA directed therapy, the ORR was 50% (n=13/26). Teclistamab responders had a higher CD8^+^:CD4^+^ T cell ratio compared to non-responders (p = 0.0176) and patients responding to teclistamab had a higher fraction of CD8^+^CD45RO^+^CCR7^-^CD62L^-^ T effector memory cells (6.2-fold increase, p = 0.0498) and CD8^+^CD45RA^+^CCR7^-^CD62L^-^ effector memory re-expressing CD45RA cells (6.7-fold increase, p = 0.0115) in their peripheral blood compared to non-responders. Additionally, patients failing to respond to teclistamab had a higher CD4^+^CD25^hi^CD127^low^ regulatory T cell population (3-fold increase, p = 0.028) with high TIGIT expression, but relatively lower expression of other inhibitory/exhaustion markers and a higher proportion of CD4^+^CD45RO^+^CCR7^+^ central memory T cells were seen in non-responders (2.4-fold increase, p = 0.003), with a subset of these cells co-expressing elevated levels of both TIGIT and PD-1, suggestive of an exhausted phenotype (31).

### Elranatamab

Elranatamab is a humanized bispecific IgG2 antibody targeting BCMA on malignant plasma cells and CD3 on T cells (32). In the phase I MagnetisMM-1 trial (NCT03269136), 101 patients were enrolled and 88 received elranatamab monotherapy either intravenously (n = 23) or subcutaneously (n = 65). Primary endpoints of the study included the incidence of dose-limiting toxicities as well as ORR and DOR. For the subcutaneous monotherapy patients, 55 patients received elranatamab at efficacious dose levels ≥215 µg kg^−1^. These 55 patients had received a median of five prior anti-myeloma therapies, 50 (90.9%) patients had triple-class refractory myeloma, 32 (58.2%) patients had penta-drug refractory myeloma and a total of 13 (23.6%) patients had received prior BCMA-targeted therapy, including 4 (7.3%) patients who had received BCMA-directed antibody drug conjugate, 5 (9.1%) patients who had received prior anti-BCMA CAR-T and four (7.3%) patients who had received both. Hematologic treatment-related adverse events included neutropenia in 41 (74.5%), anemia in 37 (67.3%), and thrombocytopenia in 28 (50.9%) patients. Non-hematologic treatment-related adverse events were CRS in 48 (87.3%) patients and injection site reaction in 31 (56.4%) patients. CRS was limited to grades 1 and 2, no grade ≥3 events were observed. ICANS was limited to grade 1 in four (7.3%) patients and grade 2 in five (9.1%) patients; no grade ≥3 ICANS events were observed. Infections of any etiology (including fungal, viral and bacterial) or grade were reported in 41 (74.5%) patients, with grade 3 events in 12 (21.8%) and grade 4 events in 3 (5.5%) patients. For the 55 patients treated with elranatamab at dose levels ≥215 µg kg^−1^, the median duration of follow-up was 12 months and the ORR was 63.6% in the entire cohort and among the 13 patients who had received prior BCMA-directed therapy, the ORR was 53.8% (n=7). A total of 13 patients with confirmed CR were MRD evaluable and all 13 patients achieved MRD negativity at a sensitivity of 1 × 10^−5^.The median DOR was 17.1 months, the median PFS was 11.8 months, and the median OS was 21.2 months. The RP2D of elranatamab was determined to be 1,000 µg kg^−1^ or 76mg once weekly (33).

In the phase II MagnetisMM-3 trial (NCT04649359), 123 patients from cohort A (BCMA-naïve) with RRMM received subcutaneous elranatamab at the RP2D of 76mg once weekly in 28-day cycles after two step-up priming doses of 12 mg and 32 mg given on day 1 and day 4 of cycle 1. After six cycles, patients who had achieved ≥PR lasting at least 2 months switched to a dosing interval of once every 2 weeks (Q2W dosing). The primary objective of the study was ORR. Patients had received a median of 5 prior lines of therapy, 96.7% had triple-class refractory disease and 42.3% had penta-drug refractory disease. After a median follow-up of 14.7 months, the primary endpoint was met with an ORR of 61.0%, with a ≥CR rate of 35.0% and of the 29 patients evaluable for MRD, 89.7% (60.5% of patients with ≥CR) of patients achieved MRD negativity to 10^−5^. The median PFS was not reached with an estimated PFS at 15 months of 50.9%. The median duration of OS was not reached with an estimated OS of 15 months of 56.7%. Infections occurred in 69.9% of patients; 39.8% had grade 3 or 4 events and 6.5% had fatal infections. CRS occurred in 56.3% of patients. All CRS events were grade 1 (42.0%) or grade 2 (14.3%), and no ≥grade 3 events were reported. ICANS occurred in 4 of 119 (3.4%) patients, with all events grade 1 or 2. With Q2W dosing, grade 3–4 adverse events decreased from 58.6% to 46.6% (34). Results from cohort B (BCMA-exposed) of the MagnetisMM-3 trial are eagerly awaited.

The phase III MagnetisMM-5 trial (NCT05020236) will evaluate the safety and efficacy of combining elranatamab with subcutaneous (SQ) daratumumab and will compare single agent elranatamab to elranatamab plus daratumumab and to daratumumab-pomalidomide-dexamethasone in patients who have received ≥3 prior lines of anti-MM therapy and are BCMA-naïve. Twenty-eight patients have been enrolled and treated in the safety lead-in cohort (part 1) which is evaluating the safety of combining SQ daratumumab with elranatamab. The most common (≥20%) treatment-related adverse events included CRS (50%; all grade 1-2), neutropenia (29%; 28% ≥grade 3), and pyrexia (21%; all G1). No patient experienced ICANS and no dose-limiting toxicities (DLT) were observed (35). Enrollment in MagnetisMM-5 is ongoing. Other studies evaluating elranatamab vs. other anti-myeloma therapies or in combination with or other anti-myeloma therapies in both newly diagnosed MM and RRMM are underway (**Table 2**).

**Table 2:** Ongoing clinical trials evaluating elranatamab in multiple myeloma.

Clinical Trials.Gov Identifier	Clinical Trial Name	Phase	Estimated Enrollment	Population	Treatment	Primary Endpoint
NCT05090566	MagnetisMM-4	Ib/II	105	RRMM	Sub-study A (SSA): elranatamab + nirogacestat Sub-study B (SSB): elranatamab + lenalidomide + dexamethasone.	Dose Limiting Toxicity (Phase I SSA) ORR (Phase II SSA) Safety and Tolerability (Dose Escalation part of SSB) Safety (Dose expansion part of SSB)
NCT05020236	MagnetisMM-5	III	762	RRMM	Elranatamab Vs. Elranatamab + Daratumumab Vs. Daratumumab+ Pomalidomide + Dexamethasone	Safety (Part 1) PFS (Part 2)
NCT05623020	MagnetisMM-6	III	966	NDMM Transplant Ineligible	Elranatamab + Daratumumab + Lenalidomide Vs. Daratumumab + Lenalidomide + Dexamethasone	Safety and RP3D (Part 1) PFS and MRD negativity at 12 months (Part 2)
NCT05317416	MagnetisMM-7	III	760	NDMM	Elranatamab Vs. Lenalidomide As post-transplant maintenance	PFS
NCT05014412	MagnetisMM-9	I/II	86	RRMM	Part 1: Patients and 2 step-up priming doses on cycle 1 D1 (4 mg) and D4 (20 mg), followed by elranatamab 76 mg QW Part 2, higher doses of elranatamab (> 76 mg) at increased intervals between doses will be evaluated	Grade ≥2 CRS rate during Cycle 1
NCT05675449	MagnetisMM-20	Ib	14	RRMM	Elranatamab + Carfilzomib + Dexamethasone And Elranatamab + Maplirpacept	Dose-Limiting Toxicity

ASCT, Autologous Stem Cell Transplant; CRS, Cytokine Release Syndrome; MRD, Minimal Residual Disease; NDMM, Newly Diagnosed Multiple Myeloma; RRMM, Relapsed Refractory Multiple Myeloma; PFS, Progression Free Survival.

### Linvoseltamab

Linvoseltamab (REGN5458) is a fully human BCMA×CD3 bispecific antibody that targets BCMA on plasma cells and CD3 on T cells. The phase I/II LINKER-MM1 trial (NCT03761108) is evaluating linvoseltamab in patients with RRMM. Treatment with linvoseltamab consists of weekly doses followed by a maintenance phase administered every 2 weeks. In the initial reports of the phase I trial, 45 patients were treated with linvoseltamab. Patients had received a median of 5 prior lines of therapy; 6.7% of patients were triple-class refractory and 53.3% were penta-drug refractory. The most common treatment-related adverse events included CRS (37.8%), fatigue (17.8%), nausea (17.8%), and myalgias (13.3%). CRS was grade 1 in 88.2% of patients and no patients had ≥grade 3 CRS. Infections occurred in 46.7% of patients with ≥ grade 3 infections occurring in 20% of patients. One patient has grade 3 syncope. The ORR was 35.6% across all dose levels (60% in highest dose level), with 31.3% of patients having ≥ CR (36). In the most recent update of this trial, 252 patients had enrolled; 73 patients in phase I and 179 patients in phase II with 75 patients having received 200mg and 104 patients having received 50mg of linvoseltamab. Patients had received a median of 5 prior lines of therapy with 81% being triple class refractory. A numerically higher efficacy was observed in patients who received 200mg of linvoseltamab having achieved an ORR of 64% compared to 50% for patients who received 50mg. The most common treatment-related adverse events at the 200mg dose were CRS (37%; grade 3: 1%), fatigue (32%; ≥grade 3: 0], and anemia (28%; ≥grade 3: 24%) and the common treatment-related adverse events at the 50mg dose were CRS (53%; grade 3: 2%), fatigue (33%; ≥grade 3: 0], and anemia (40%; ≥grade 3: 36% (37). Longer follow-up from the 200mg cohort is available; as of February 28, 2023, 117 RRMM patients enrolled into the 200 mg cohort with 74% of patients being triple-class refractory. The median duration of follow-up was 5.6 months, and the ORR was 71% with ≥CR rate of 30% with ORR and ≥CR rates being 70% and 29% in triple-class refractory patients, respectively. The probability of PFS at 12 months was 66% in the entire cohort. The most common treatment-related adverse events were CRS (45%; grade 3-4:1%), cough (33%; grade 3-4: 0%), neutropenia (32%: grade 3-4: 31%), diarrhea (32%; grade 3-4: 2%), and fatigue (32%; grade 3-4: 0%). Rate of infections of any grade was 59.8% with ≥grade 3 in 36.8% of patients (38). **Table 3** shows ongoing clinical trials evaluating linvoseltamab single-agent and in combination with other anti-myeloma therapies in patients with newly diagnosed myeloma and RRMM.

**Table 3:** Ongoing trials with other BCMA-targeting bispecific antibodies.

Clinical Trials.Gov Identifier	Clinical Trial Name	Phase	Estimated Enrollment	Population	Treatment	Primary Endpoint
Linvoseltamab						
NCT03761108	LINKER-MM1	I/II	387	RRMM	Linvoseltamab	Safety (Phase I) ORR (Phase II)
NCT05137054	–	I	317	RRMM	• Linvoseltamab + Daratumumab • Linvoseltamab + Carfilzomib • Linvoseltamab + Lenalidomide • Linvoseltamab + Bortezomib • Linvoseltamab + Pomalidomide • Linvoseltamab + Isatuximab • Linvoseltamab + Fianlimab • Linvoseltamab + Cemiplimab • Linvoseltamab + Nirogacestat	Dose-Limiting Toxicity and Safety
NCT05730036	LINKER-MM3	III	286	RRMM	Linvoseltamab Vs. Elotuzumab + Pomalidomide + Dexamethasone	PFS
NCT05828511	LINKER-MM4	I/II	132	NDMM	Linvoseltamab	Dose-Limiting Toxicity and Safety (Phase I) ≥VGPR rate + MRD Negativity Rate (10^-5^)
ABBV-383						
NCT05650632	–	I	120	RRMM	ABBV-383	Grade ≥ 2 CRS Rate
NCT06223516	–	I	55	RRMM	ABBV-383 (SQ)	Safety and Pharmacodynamic of subcutaneous ABV-383
NCT05259839	–	I	270	RRMM	• ABBV-383 + Pomalidomide + Dexamethasone • ABBV-383+ Lenalidomide + Dexamethasone • ABBV-383 + Daratumumab +Dexamethasone • ABBV-383 + Nirogacestat	Dose Limiting Toxicities
NCT06158841	–	III	380	RRMM	ABBV-383 Vs. Carfilzomib + Dexamethasone Or Elotuzumab + Pomalidomide + Dexamethasone Or Selinexor + Bortezomib + Dexamethasone	PFS and ≥VGPR rate
Alnuctamab						
NCT06163898	–	I/II	156	RRMM	Alnuctamab + Mezigdomide	Dose-Limiting Toxicities and Safety (Phase I) ORR (Phase II)
NCT06232707	ALUMMINATE	III	466	RRMM	Alnuctamab Vs. Daratumumab + Pomalidomide + Dexamethasone Or Carfilzomib + Dexamethasone Or Elotuzumab + Pomalidomide + Dexamethasone	PFS
NCT06121843	0	I	111	RRMM	• CC-95266 • Alnuctamab + Mezigdomide • Alnuctamab + Iberdomide	Safety and RP2D

CRS, Cytokine Release Syndrome; MRD, Minimal Residual Disease; NDMM, Newly Diagnosed Multiple Myeloma; RRMM, Relapsed Refractory Multiple Myeloma; ORR, Overall Response Rate; PFS, Progression Free Survival; RP2D, Recommended Phase 2 Dose; VGPR, Very Good Partial Response.

### ABBV-383

ABBV-383 (formerly TNB-383B) is a fully human, monoclonal, IgG4 BCMA × CD3 bispecific antibody with 2 BCMA-binding domains and a low-affinity CD3-binding domain to potentially mitigate the incidence of CRS and eliminate the need for a step-up dosing schedule (39, 40). In a phase 1 (NCT03933735) dose escalation/dose expansion trial evaluating single agent ABBV-383 in patients with RRMM, ABBV-383 was administered IV over 1-2 hours at a fixed dose once every 3 weeks in the dose escalation phase and 14 different dose levels were evaluated ranging from 0.025 mg to 120mg. No step-up doses were implemented. As of January 8, 2022, 124 patients were enrolled (escalation phase, n = 73; expansion phase, n = 51) and had received a median of 5 prior lines of therapy; 82% (n=102) of patients were triple-class refractory and 35% (n=44) of patients were penta-drug refractory. At the time of the data cutoff, enrollment of the 60 mg expansion cohort had been completed, and the protocol was in process of being amended to investigate a lower dose (40 mg once every 3 weeks) in an additional expansion cohort. The most common (≥ 25%) treatment-related hematologic adverse events were neutropenia (37%; ≥grade 3: 34%) and anemia (29%; ≥grade 3: 16%) with a similar adverse event reported for the ≥ 40 mg escalation plus expansion and 60 mg expansion cohorts. The most common nonhematologic treatment-related adverse events in the overall population were CRS (57%), fatigue (30%), nausea (29%), and diarrhea (27%). Grade ≥ 3 infections in the overall population were pneumonia, sepsis, COVID-19 disease (6% each), and urinary tract infections (5%). The ORR and ≥ VGPR rates for all evaluable patients (n = 122) were 57% and 43%, respectively. ICANS occurred in 2 patients at the 60mg dose. Amongst the 49 patients in the 60 mg expansion cohort, the ORR and ≥ VGPR rates were 59% and 39%, respectively. Among the 79 patients in the ≥ 40 mg escalation plus expansion cohorts ORR and ≥ VGPR rate were 68% and 54%, respectively. Of the 11 MRD-evaluable patients with ≥CR, 8 (73%) were MRD-negative (≤ 10^–5^). In triple-class refractory patients (n = 100), the ORR was 51%. After a median follow-up of 10.8 months in the overall population, the median PFS was 10.4 months (41).

In an update of NCT03933735, 220 patients were treated with IV ABBV-383 as of May 17, 2023; 73 patients in the dose escalation cohort and 147 in the dose expansion with 32 patients at 20 mg, 55 patients at 40 mg, and 61 patients at 60 mg Q3W. Patients had received a median of 5 prior lines therapy and 80% were triple-class refractory. CRS was the most common treatment-related adverse event and it occurred in 71% (45% grade 1, 25% grade 2, and 0% ≥grade 3) of patients at 40 mg, and 70% (51% grade 1, 18% grade 2, and 2% grade 3) of patients at 60mg. ICANS occurred in 5% (2% grade 1, 4% grade 2, and 0% ≥grade 3) of patients at 40 mg, and 5% (3% grade 2 and 2% grade 3) of patients at 60 mg. No ≥grade 4 CRS or ICANS occurred. Infections occurred in 71% (26% ≥grade 3) of patients at 40 mg, and 57% (34% ≥grade 3) of patients at 60 mg. Duration of response estimates at 12 months were 70% at 40 mg and 66% at 60 mg. The median PFS was 13.7 months at 40 mg, and 11.2 months at 60 mg. The median OS was not reached at 40 mg or 60 mg (42). Ongoing clinical trials evaluating ABBV-338 in RRMM are shown in **Table 3**.

### Alnuctamab

Alnuctamab is a symmetric 2-arm humanized IgG bispecific antibody that binds bivalently to BCMA and monovalently to CD3 in a 2 + 1 format (43). NCT03486067 is a phase 1 dose-finding study evaluating alnuctamab in patients with RRMM. Initially, patients on the trial received IV-administered alnuctamab and demonstrated durable responses (median duration of response, 33.6 months) but due to a high frequency of CRS in 76% of patients (including grade ≥ 3 events in 7% of patients), the trial turned to SQ administration of alnuctamab. Of 73 patients treated with SQ alnuctamab in dose escalation (target dose: 10 mg, 15 mg, 30 mg, and 60 mg) and dose expansion (target dose: 10 mg 30 mg, and 60 mg), patients had received a median of 4 prior lines of therapy and 63% were triple-class refractory and 19% were penta-drug refractory. The most common treatment related adverse events were CRS (56%; ≥grade 3: 0%), neutropenia (55%; ≥grade 3: 45%), anemia (47; ≥grade 3: 27%), and thrombocytopenia (37%; ≥grade 3: 16%). All-grade and grade ≥ 3 infections occurred in 62% and 16% of patients, respectively. Two patients had grade 1 ICANS suspected to be related to alnuctamab; no grade ≥ 2 ICANS was observed. ORR in the entire cohort was 54% and ORR was 63% (n=27/43) at target doses ≥ 30 mg and 69% (n=18/26) at the 30-mg target dose. Among all patients, the median PFS was 10.1 months. Among the 39 patients who achieved a response, 100% (n=28/28) with evaluable MRD samples were MRD-negative (10 ^−5^) (44). Ongoing clinical trials evaluating alnuctamab in RRMM are shown in **Table 3**.

### WVT078

WVT078 is a novel bispecific antibody with a human IgG1 backbone that binds to BCMA on myeloma cells and on T cells. The IgG1- based antibody backbone of WVT078 differs from that of other BCMA × CD3 bispecific antibodies including teclistamab (IgG4-based) and elranatamab (IgG2-based) (45). A phase 1 dose- escalation study of WVT078 as a single agent and in combination with a gamma-secretase inhibitor is ongoing (NCT04123418). As of March 07, 2022, 33 patients with RRMM have been treated with various doses (3-250 µg kg weekly) of IV WVT078 with 51.5% (n=17) and 18.2% (n=6) being triple-class and penta-drug refractory, respectively. The most frequent (≥20%) all-grade treatment-related adverse events included CRS (60.6%), pyrexia (39.4%), increased alanine aminotransferase (30.3%), anemia (24.2%) and increased aspartate aminotransferase (24.2%). Grade ≥3 treatment-related adverse events occurred in 51.5% (n=17) of patients with the most common (≥10%) being increase in aspartate aminotransferase (21.2%), lymphopenia (18.2%), increase in alanine aminotransferase (15.2%), CRS (12.1%), and neutropenia, (12.1%). There were no reports of ICANS or other neurotoxicity. Fifty-eight percent (n=19) of patients had infections of any grade on study with WVT078. Four (12%) infection events were grade 3 and there were no grade 4 infections. Clinical activity began at the 48 µg/kg dose of WVT078 and at doses between 48–250 µg/kg (n = 26), the ORR was 38.5%. At the highest dose level (250 µg/kg), the ORR was 75% (n=3/4). The median duration of response was 7.6 months (45). WVT078 is being evaluated as part of the phase I trial in combination with the gamma secretase inhibitor WHG626 (AL102). As of April 29, 2023, a total of 23 patients with RRMM were treated in dose escalation with IV WVT078 at 12-48 µg/kg once weekly, combined with oral WHG626 at 2-4 mg once daily, given 2 days on and 5 days off. The most frequent (≥20%) treatment-related adverse events across all doses in the combination were CRS (65.2%; Grade ≥3, n=1), pyrexia (39.1%), diarrhea (34.8%), decreased appetite (26.1%), hypophosphatemia (21.75%), nausea (21.7%) and neutropenia (21.7%); 5 patients experienced dose-limiting toxicities. The ORR and ≥CR rate across all dose levels tested were 39.1% and 13.0%, respectively. At the highest dose levels tested, the ORRs were 57.1% (46). Enrollment in NCT04123418 is ongoing.

## GPRC5d-directed bispecific antibodies

G protein–coupled receptor, class C, group 5, member D (GPRC5d) is an orphan G protein–coupled receptor whose expression is limited to two anatomic locations: the hair follicle and the bone marrow of patients with MM (47–49). Studies have confirmed that GPRC5d is consistently expressed on MM cells with a membranous pattern and is absent from nearly all healthy tissues, with the exception of the hair follicle, making it an ideal target for anti-myeloma therapy (50).

### Talquetamab

Talquetamab, a bispecific IgG4 antibody that binds to both GPRC5d and CD3 to induce killing of GPRC5d-expressing myeloma cells by means of T-cell-mediated cytotoxicity (51, 52). MonumenTAL-1 (NCT04634552/NCT03399799) is a phase I/II, dose-escalation trial evaluating the safety and RP2D talquetamab in RRMM. At the data-cutoff date, 232 patients had received talquetamab administered IV weekly or every other week (at dose ranging from 0.5 to 180 μg/kg; n=102) or SQ weekly, every other week, or monthly (5 to 1600 μg/kg; n=130). Patients had received a median of 6 prior lines of therapy; 79% of the patients had triple-class–refractory myeloma and 30% had penta-drug–refractory myeloma. Four DLTs occurred during the dose-escalation phase; grade 4 lipase increase (IV dose), grade 3 maculopapular rash in two patients, and a grade 3 rash; all rashes occurred in patients who received SQ doses of talquetamab. After these DLTs, the DLT criteria were modified to exclude the first occurrence of a glucocorticoid-responsive grade 3 rash that began to resolve within 7 days after treatment. SQ talquetamab was associated with a more favorable pharmacokinetic profile and the 405-μg dose level (with step-up doses of 10 and 60 μg per kilogram) and the 800-μg dose level (with step-up doses of 10, 60, and 300 μg per kilogram) were chosen for confirmation in part 2 of the study. Thirty patients received SQ talquetamab at the 405-μg dose level (median follow-up 11.7 months) and 44 patients received SQ talquetamab at the 800-μg dose level (median follow-up 4.2 months). Common adverse events at the 405- μg week dose (n=30) included CRS (77%; ≥grade 3: 3%), skin-related (67%; ≥grade 3: 0%), neutropenia (67%; ≥grade 3: 60%), dysgeusia (63%; ≥grade 3: N/A), anemia (60%; ≥grade 3: 30%), nail-related (57% ≥grade 3: 0%) and rash-related events (47%; ≥grade 3: 0%). Common adverse events at the 800-μg every 2 week dose (n=44) included CRS (80%; ≥grade 3: 0%), skin-related (70%; ≥grade 3: 2%), dysgeusia (57%; ≥grade 3: N/A), anemia (43%; ≥grade 3: 23%), neutropenia (36%; ≥grade 3: 60%), rash-related events (30%; ≥grade 3: 16%), and nail-related (27% ≥grade 3: 2%). Infections occurred in 47% of the patients who had received talquetamab at the 405-μg dose level (≥grade 3: 7%) and in 34% of those who had received it at the 800-μg dose level (≥grade 3: 7%). Among patients who received talquetamab at the 405-μg dose level, the ORR was 70% (≥CR: 23%) and was 64% (≥CR: 23%) among patients who received talquetamab at the 800-μg dose level. The median DOR was 10.2 months in patients who received talquetamab at the 405-μg dose level and 7.8 months in those who received it at the 800-μg dose level. Amongst the patients with triple-class–refractory disease, the ORR was 65% and 70% at the 405-μg and 800-μg doses, respectively. Among patients who received prior BCMA-directed bispecific antibody or CAR-T (n=16), the ORR was 50% (53). Based on the results of the MonumenTAL-1 study, the FDA approved talquetamab for patients with RRMM after ≥ 4 prior lines of therapy on August 9, 2023.

In the phase II portion of the MonumenTAL-1 study, 288 patients received SQ talquetamab; 143 received talquetamab 0.4 mg/kg every week (QW) and 45 received talquetamab 0.8 mg/kg every 2 weeks (Q2W). Common adverse events included CRS (79% [QW], 75% [Q2W]), skin-related (56% [QW], 71% [Q2W]), nail-related (54% [QW], 53% Q2W])), and dysgeusia (50% [QW], 48% [Q2W]). Most of these adverse events were grade 1 or grade 2. ICANS occurred in 11% of patients in both dosing cohorts and infections occurred in 58% (≥grade 3: 22%) and 65% (≥grade 3: 16%) in the QW and Q2W dosing cohorts, respectively. The ORR was 49% in both cohorts and the median PFS was 7.5 months in the QW cohort and 11.9 months in the Q2W dosing cohort (54). Across the phase I and phase II portions of the MonumenTAL-1 study, 70 patients (as of January 17, 2023) had received prior bispecific antibody or CAR-T cell therapy. Eighty-three percent and 41% were triple-class refractory and penta-drug refractory, respectively. Fifty of 70 patients (71%) had received prior CAR-T (48/50 had received prior anti-BCMA CAR-T), 25 (36%) patients received prior bispecific antibodies (23/25 had received prior anti-BCMA bispecific antibody), and 5 (7%) patients received both. Eight of 70 (11%) patients received prior treatment with an anti-BCMA antibody drug conjugate. The ORR was 65.7% amongst the 70 patients who had received prior CAR-T or bispecific antibody; the ORR was 72.9% (n=35/48) amongst those who had received prior anti-BCMA CAR-T, 52.2% (n=12/23) amongst those who had received prior anti-BCMA bispecific antibody, and 75% (n=6/8) amongst those who had received prior anti-BCMA antibody drug conjugate and either anti-BCMA bispecific anybody or anti-BCMA CAR-T (55). These results showed the efficacy of talquetamab in the post-BCMA directed therapy setting.

The phase I MonumenTAL-1 study had cohorts that evaluated reduced dose intensity of talquetamab. In one cohort, patients who were treated with talquetamab 0.8 mg/kg every 2 weeks were permitted to switch to 0.4 mg/kg every two weeks following a confirmed ≥PR. In another cohort, patients who were being treated with talquetamab 0.8 mg/kg every two weeks were permitted to switch to 0.8 mg/kg every 4 weeks following a confirmed ≥PR. Forty-five patients switched to reduced intensity dosing and as of June 20, 2023, 24 patients were in the analysis with a median follow-up of 9.7 months. In total, 75% (n=9/12) of patients achieved a ≥PR and switched from 0.8 mg/kg every 2 weeks to 0.4 mg/kg every 2 weeks dosing, and 83% (n=10/12) of patients achieved a ≥PR and switched from every 2 week to 0.8 mg/kg every four-week dosing. Responses deepened in 57.9% (n=11/19) of patients and were maintained in 26.3% (n=5/19) of patients. Three of 19 patients had disease progression. Oral-related treatment-related adverse events, reported in 84.2% (n=16/19) of patients, improved or resolved in 4 patients (21%) after switching to reduced intensity dosing. Nail-related treatment-related adverse events, reported in 36.8% (n=7/19) of patients, improved or resolved in 2 patients. Skin-related treatment-related adverse events, reported in 42.1% (n=8/19) of patients, resolved in 3 patients (56). These data suggest that reduced dose intensity of talquetamab may mitigate its side effect profile with regards to skin, oral and nail-related adverse events.

MonumenTAL-2 (NCT05050097) is an ongoing multi-arm, phase 1b study of talquetamab in combination with anti-myeloma agents (pomalidomide, lenalidomide, lenalidomide + daratumumab, carfilzomib and carfilzomib + daratumumab) in patients with RRMM. The cohort evaluating talquetamab in combination with pomalidomide has reported results. As of June 5, 2023, 35 patients were enrolled and received the RP2D of subcutaneous talquetamab; 0.4 mg/kg weekly or 0.8 mg/kg every other week (with step-up dosing) plus oral pomalidomide 2 mg daily (dose escalation to 4 mg daily permitted) starting in cycle 2. Patients had received a median of 3 prior lines of therapy in both cohorts and 25% and 21% were triple-class refractory in the weekly and every other week talquetamab dosing cohorts, respectively. The most common treatment-related adverse events were dysgeusia (77.1%), CRS (74.3%; 2.9% ≥grade 3), and neutropenia (60.0%). The most common ≥grade 3 treatment-related adverse events were neutropenia (48.6%), anemia (25.7%), and thrombocytopenia (20.0%). Nail (65.7%), skin (40%) and rash (20%) adverse events were mostly grade 1 or 2. Two patients experience grade 1 ICANS and infections occurred in 71.4% of patients with 22.9% being ≥ grade 3. The ORR was 86.7% (≥CR: 60%) and 83.3% (≥CR: 44.4%) in the weekly and every other week cohorts, respectively. The median DOR and PFS were not reached, with 6-month PFS rates of 93.3% and 88.9% in the weekly and every other week cohorts, respectively (57).

In the phase I TRIMM-2 (NCT04108195) study, the cohort evaluating talquetamab in combination with daratumumab has reported results. As of Dec 12, 2022, 65 patients received talquetamab at the RP2Ds with step-up dosing in combination with standard dosing of SQ daratumumab (1800 mg). Patients had received a median of 5 prior lines of therapy; 58% were triple-class refractory and 63% were penta-drug exposed. Prior treatments included anti-BCMA-directed therapy in 54% of patients including 25% who had received prior anti-BCMA directed bispecific antibody and 17% who had received anti-BCMA directed CAR-T. The most common treatment-related adverse events were CRS (78%; all grade 1/2), dysgeusia (75%), dry mouth (55%), anemia (52%), fatigue (45%), and skin exfoliation (45%). ICANS occurred in 3 patients (5%) and was either grade 1 or 2. The ORR was 78% (45% ≥CR) and the ORRs in patients refractory to daratumumab, anti-BCMA directed therapy and bispecific antibody therapy were 76%, 64%, and 75%, respectively. After a median follow up was 11.5 months, the median PFS was 19.4 months and the 12-month PFS and OS rates were 76% and 93%, respectively (58). Upregulation of proinflammatory cytokines and CD38+/CD8+ T cells was observed with talquetamab + daratumumab, supporting the synergy of this combination in patients with prior anti-CD38 exposure (59).

In the initial reported results of the phase 1b RedirecTT-1 trial (NCT04586426), 63 patients (as of Dec 12, 2022) were treated with teclistamab plus talquetamab. Patients had received a median of 5 prior lines of therapy, 78% were triple-class refractory, and 43% had extramedullary disease (EMD). The most common treatment-related adverse events were CRS (81%; grade 3: 3%), neutropenia (76%; ≥grade 3: 75%), and anemia (60%;≥grade 3: 43%). One ICANS event was reported at dose level 3 and no DLTs were reported at the recommended phase 2 regimen (RP2R). Across all dose levels, the ORR was 84% (n=52/62) and the ≥CR rate was 34% (n=21/62). At the RP2R, the ORR was 92% (n=12/13; ≥CR: 31%) among all evaluable patients and 83% (n=5/6; ≥CR: 33%) amongst patient with EMD. After a median follow-up of 14.4 months, the median DOR has not been reached (60).

Ongoing clinical trials evaluating talquetamab in newly diagnosed MM and RRMM and in combination with other agents are shown in **Table 4**.

**Table 4:** Ongoing clinical trials evaluating talquetamab in multiple myeloma.

Clinical Trials.Gov Identifier	Clinical Trial Name	Phase	Estimated Enrollment	Population	Treatment	Primary Endpoint
NCT05050097	MonumenTAL-2	Ib	182	RRMM	Talquetamab in combination with: • Carfilzomib • Carfilzomib + Daratumumab • Lenalidomide • Lenalidomide + Daratumumab • Pomalidomide	Safety and Dose Limiting Toxicities
NCT05455320	MonumenTAL-3	III	810	RRMM	Talquetamab + Daratumumab Vs. Talquetamab + Daratumumab + Pomalidomide Vs. Daratumumab + Pomalidomide + Dexamethasone	PFS
NCT06208150	MonumenTAL-6	III	795	RRMM	Talquetamab + Pomalidomide Or Talquetamab + Teclistamab Vs. Elotuzumab + Pomalidomide + Dexamethasone Or Bortezomib + Pomalidomide + Dexamethasone	PFS
NCT05338775	TRIMM-3	I	152	RRMM	Talquetamab + PD-1 inhibitor Or Teclistamab + PD-1 inhibitor	Safety and Dose Limiting Toxicities
NCT04586426	RedirecTT-1	I/II	164	RRMM	Talquetamab + Teclistamab Or Talquetamab + Teclistamab + Daratumumab	Safety, Dose Limiting Toxicity and ORR
NCT04108195	TRIMM-2	I	289	RRMM	Daratumumab + Teclistamab Or Daratumumab + Talquetamab Or Daratumumab + Talquetamab + Pomalidomide Or Daratumumab + Teclistamab + Pomalidomide	Safety and Dose Limiting Toxicities
NCT06066346	–	II	17	RRMM	Talquetamab in the post- anti-BCMA CAR-T setting	ORR
NCT05552222	MajesTEC-7	III	1590	NDMM (Transplant Ineligible)	Teclistamab + Daratumumab + Lenalidomide Vs. Talquetamab + Daratumumab + Lenalidomide Vs. Daratumumab + Lenalidomide + Dexamethasone	PFS
NCT05849610	GEM-TECTAL	II	30	High Risk NDMM	NDMM patients with high-risk cytogenetics will receive quadruplet induction therapy with Dara-VRD followed by intensification with Teclistamab + Daratumumab and patients who are MRD+ after intensification will receive: Talquetamab +Daratumumab	MRD Negativity Rate

Dara-VRD, Daratumumab-Bortezomib-Lenalidomide-Dexamethasone; MRD, Minimal Residual Disease; NDMM, Newly Diagnosed Multiple Myeloma; RRMM, Relapsed Refractory Multiple Myeloma; ORR, Overall Response Rate; PFS, Progression Free Survival.

### Forimtamig

Forimtamig is a GPRC5d×CD3 -directed bispecific antibody with a novel 2:1 design that confers bivalent binding to GPRC5d and increased T-cell directed killing compared with other molecular formats (61). A phase I trial (NCT04557150) is evaluating forimtamig in patients with RRMM. As of June 8, 2022, 51 patients had been enrolled in the IV forimtamig cohorts and 54 patients into the SQ cohorts. Patients were treated with a median of 5 and 4 prior lines of therapy in the IV and SQ cohorts, respectively. Sixty-three percent were triple-class refractory in the IV cohorts and 73.1% in the SQ cohorts and 30.6% were penta-refractory in the IV cohort and 42.3% in the SQ cohorts. About 20% of patients in both the IV and SQ cohorts had received prior anti-BCMA -directed therapy. CRS was the most common adverse event (IV: 82.4%; SC: 77.8%; about 2% in each cohort was ≥grade 3). ICANS occurred in 8.6% (n=9) of patients and with only 2 patients experiencing ≥grade 3 ICANS. Other AEs included dermal and epidermal AEs (IV: 72.5%; ≥grade 3: 11.8%; SQ: 81.5%; ≥grade 3: 14.8%), hair and nail changes (IV: 17.6%; SQ: 22.2%: all were grade 1 or 2), and oral AEs (IV: 70.6% [all grade 1-2]; SQ: 74.1%; grade 3: 5.6%). Infectious AEs were common (IV: 56.9%; ≥3 grade 3: 19.6%; SQ: 37.0%; ≥ grade 3: 24.1%). The ORR was 71.4% in the IV cohorts and 60.4% in the SQ cohorts. Responses were observed in 55.6% (n=10/18) of patients across IV and SQ cohorts who had received prior anti-BCMA therapies. After a median follow-up time of 7.1 months in the IV cohorts and 3.9 months in the SQ cohorts, the duration of follow-up data was immature at data cut-off (62).

## FcRH5-directed bispecific antibodies

Fc receptor-homolog 5 (FcRH5) is a is a cell surface antigen of unknown function whose expression is restricted to mature B cells and compared to normal human plasma cells, FCRH5 is expressed at higher levels in malignant plasma cells (63, 64).

### Cevostamab

Cevostamab is an IgG-based bispecific antibody that targets the membrane-proximal domain of FcRH5 on malignant plasma cells and CD3 on T cells (64). In the dose-escalation portion of the phase I (NCT03275103) trial evaluating cevostamab in RRMM, patients received IV cevostamab in 21-day cycles; the single step-up cohort received step-up doses (0.05-3.6mg) given on cycle 1 day 1 and the target dose (0.15-198mg) on cycle 1 day 8 and in the double step-up cohorts, the step doses were given on cycle 1 day 1 (0.3-1.2mg) and cycle 1 day 8 (3.6mg), and the target dose (60-160mg) on cycle 1 day 15. As of May 18, 2021, 160 patients have been enrolled. Patients had received a median of 6 prior lines of therapy, 85% of were triple-class refractory, and 17.5% (n=28) had received ≥1 prior CAR-T, 8.1% (n=13) had received ≥1 prior bispecific antibody, 16.9% (n=27) had received ≥1 prior antibody-drug conjugate, and 33.8% (n=54) had received ≥1 prior anti-BCMA targeting agent. The most common treatment-related adverse events were CRS (80.0%; grade 3: 1.3%), infections (80%; ≥grade 3: 18.8%), anemia (31.9%; ≥grade 3: 21.9%), and diarrhea (26.3% grade 3: 0.6%). ICANS events were observed in 13.1% (grade 3: 1.4%) of patients. In the dose-escalation phase, responses were observed at the 20-198mg target dose levels and two dose-expansion cohorts were opened, 90mg and 160mg. The ORR was higher at the 160mg dose level at 54.5% (n=24/44) compared to the 90mg dose level at 36.7% (n=22/60). At dose levels >90mg, the ORR in patients with prior exposure to CAR-Ts, bispecific antibodies, antibody drug conjugates, and anti-BCMA targeting agents were 44.4% (n=4/9), 33.3% (n=3/9), 50.0% (n=7/14), and 36.4% (n=8/22), respectively. After a median follow-up of 8.1 months among responders; the estimated median duration of response was 15.6 months (65). In patients pre-treated with tocilizumab prior to receiving cevostamab, the overall rate of CRS was significantly lower in the tocilizumab group than in the non-tocilizumab group (38.7% vs 90.9%, respectively). There was no negative impact on the anti-MM activity of cevostamab amongst patients who received tocilizumab. The ORRs were 54.8% and 37.2% in the tocilizumab and non-tocilizumab groups, respectively (66). Ongoing clinical trials evaluating cevostamab in RRMM and in combination with other agents are shown in **Table 5**.

**Table 5:** Ongoing clinical trials evaluating cevostamab in multiple myeloma.

Clinical Trials.Gov Identifier	Clinical Trial Name	Phase	Estimated Enrollment	Population	Treatment	Primary Endpoint
NCT04910568	CAMMA 1	I	184	RRMM	Cevostamab Or Cevostamab + Pomalidomide + Dexamethasone Or Cevostamab + Daratumumab + Dexamethasone	RP2D and Safety
NCT05535244	CAMMA 2	I/II	90	RRMM	Cevostamab after either: • Prior BCMA ADC or CAR-T • Prior BCMA BsAb • Prior BCMA CAR-T	ORR and Adverse Events
NCT05801939	–	II	30	RRMM	Cevostamab in the Post-BCMA Setting	CR rate and MRD Negativity Rate
NCT05646836	–	I	90	RRMM	Cevostamab Or Cevostamab + XmAb24306	Percentage of Adverse Events
NCT05927571	–	I	120	RRMM	Cevostamab + Elranatamab	Percentage of Adverse Events
NCT05583617	PLYCOM	I/II	200	RRMM	Cevostamab + Lenalidomide Or Cevostamab + Iberdomide + Dexamethasone	Percentage of Adverse Events and ORR

CR, Complete Response; MRD, Minimal Residual Disease; RRMM, Relapsed Refractory Multiple Myeloma; ORR, Overall Response Rate; PFS, Progression Free Survival; RP2D, Recommended Phase 2 Dose.

## Bispecific and trispecific antibodies targeting other immune effector cells and plasma cell targets

### CD38-targeting bispecific antibodies

CD38 is highly expressed on the surface of malignant plasma cells, functions as a receptor for CD31, has ectoenzymatic activities and is already targeted with monoclonal antibodies (daratumumab and isatuximab) in patients with MM (67, 68).

### ISB 1342

ISB 1342 is a CD38 x CD3 bispecific antibody which binds to CD38 on malignant plasma cells (on a different epitope than daratumumab) and has a detuned scFv domain affinity binding to CD3 on T cells, to mitigate the risk of CRS (69). In an ongoing phase I dose-escalation trial (NCT03309111), 39 patients with RRMM received weekly dose escalations of IV or SQ ISB-1342 in 8 dose-escalation cohorts ranging from 0.2/0.3 mg/kg to 4.0/16.0 mg/kg as priming/treatment doses. An additional 7 patients had received weekly SQ injections at 2.0/8.0 mg/kg. Patients had received a median of 6 prior lines of therapy and 67% (n=21) of patients had triple-class refractory disease and 48% (n=18) had penta-drug refractory disease. Treatment-related adverse events included infusion related reactions (37%), CRS (34%, all grade 1-2), anemia (24%), neutropenia (24%), and thrombocytopenia (17%) (70).

### ISB 1442

ISB 1442 is a fully human bispecific antibody that targets CD38 and CD47 that is designed to kill CD38-expressing tumor cells through multiple immune-mediated mechanisms via the blocking of SIRPα, the CD47-signal regulatory protein which leads to increased complement-dependent cytotoxicity, antibody-dependent cellular cytotoxicity, and antibody-dependent cellular phagocytosis (71). A phase I (NCT05427812) dose-escalation study of ISB 1442 in RRMM has reported preliminary results. As of July 18, 2023, 10 patients had received weekly SQ injections of ISB 1442 in 4 dose-escalation groups ranging from 6 mg-150 mg. Patients had received a median of 6 prior lines of anti-myeloma therapy and 70% of patients were penta-drug exposed. Eight patients (80%) experienced treatment-related adverse events, all of which were grade 1 or 2: CRS (50%), and injection site reactions (30%) were the most common. This clinical trial is ongoing and continues to recruit patients (72).

Ongoing clinical trials with other CD38-targeting bispecific antibodies and other novel targets are shown in **Table 6**.

**Table 6:** Ongoing clinical trials evaluating other bispecific antibodies and trispecific antibodies in multiple myeloma.

Clinical Trials.Gov Identifier	Clinical Trial Name	Phase	Estimated Enrollment	Population	Treatment	Primary Endpoint
CD38 x CD3 Bispecific						
NCT05908396	–	I	100	RRMM	IGM-2644	Safety, tolerability and MTD
NCT03309111	–	I	245	RRMM	ISB 1342	MTD and RP2D (Part I) ORR (Part II)
CD38 x CD47 Bispecific						
NCT05427812	–	I/II	121	RRMM	ISB 1442	Safety and MTD (Phase I) ORR (Phase II)
CD1d x Vd2-T cell receptor chain Bispecific						
NCT04887259	–	I/II	102	RRMM	LAVA-051	Percentage of Adverse Events
BCMA x GPRC5D x CD3 Trispecific						
NCT05652335	–	I	170	RRMM	JNJ-79635322	DLT and Adverse Event Rate
CD38 x CD28 x CD3 Trispecific						
NCT04401020	–	I	57	RRMM	SAR442257	MTD and RP2D
BCMA x CD38 x CD3 Trispecific						
NCT05862012	–	I	80	RRMM	ISB 2001	DLT and Safety
BCMA x Albumin x CD3 Trispecific						
NCT04184050	–	I	97	RRMM	HPN217	DLT and Adverse Event Rate

DLT, Dose Limiting Toxicity; MTD, Maximum Tolerated Dose; ORR, Overall Response Rate; RP2D, Recommended Phase 2 Dose; RRMM, Relapsed Refractory Multiple Myeloma.

### Trispecific antibodies and NK-cell engagers

Trispecific antibodies, or antibodies that target two malignant plasma cell antigens and a T-cell or natural killer (NK)-cell antigen to stimulate T-cell or NK-cell cytotoxicity are in clinical development for the treatment of RRMM. By targeting two malignant plasma cell antigens, antigen escape by myeloma cells as a mechanism of resistance may be mitigated. JNJ-79635322 is a trispecific antibody composed of an anti-CD3, anti-BCMA binding domain, and anti-GPRC5d binding domain that is currently in clinical development. JNJ-79635322 showed efficacy in a murine MM xenograft model and a phase 1 dose-escalation study is ongoing (NCT05652335) (73). SAR442257 is a CD38xCD28xCD3 trispecific T-cell engager. Interaction of the trispecific antibody with CD28 provides a costimulatory signal to the T-cell and mitigates T-cell exhaustion and while treatment with anti-CD38 monoclonal antibodies like daratumumab and isatuximab lead to CD38 downregulation on malignant plasma cells and impaired NK-cell activity, SAR442257 depends less on CD38 density and NK cell function thus should be a more potent CD38-directed immunotherapy via its T-cell activation and stimulation and minimal NK-cell activity (74). A phase I trial (NCT04401020) evaluating SAR442257 in patients with RRMM is ongoing. ISB 2001 is a trispecific T-cell engager that redirects cytotoxic T cells via binding to CD3 to BCMA as well as CD38 expressing myeloma cells. This mechanism of action (dual binding to CD38 and BCMA) may overcome malignant plasma cell escape mechanisms associated with low tumor antigenic expression inherent to myeloma as preclinical work has shown that ISB 2001 demonstrates improved anti-myeloma activity when compared to other BCMA- or CD38-directed targeted therapeutic either as single agent or in combination across different MM models (75). A phase 1 study (NCT05862012) evaluating ISB 2001 in RRMM is ongoing. HPN217 is a trispecific T-cell engager that binds to BCMA on malignant plasma cells, albumin for half-life extension, and CD3 for T-cell engagement. There is an ongoing phase I (NCT04184050) trial evaluating escalating doses of HPN217 in patients with RRMM. As of June 27, 2022, 49 patients received HPN217. The highest fixed-dose level administered was 2860 μg/wk. Patients had received a median of 6 prior lines of therapy and 69% of patients were penta-drug exposed, and 22% had received prior BCMA-targeted therapy. The most common treatment-related adverse events were anemia (49%), fatigue (37%), CRS (25%), nausea (22%), transaminitis (20%), arthralgia (20%), and diarrhea (20%). All CRS events were either grade 1 or grade 2 and no ICANS events were reported. No DLTs have been reported (76). Ongoing clinical trials with trispecific antibodies in RRMM are shown in **Table 6**.

Multiple bispecific NK-cell engagers are in pre-clinical development and target NK-cell antigens such as NKG2D, CD16a, MICA, CD16a and CD200,NKp30, Nkp46 and myeloma cell antigens such as CS1, BCMA, CD38 (77–81).

## Mechanisms of resistance to bispecific antibodies and potential mechanisms to overcome resistance

Mechanisms of resistance of BMCA- and GPRC5d-targeting bispecific antibodies in RRMM to date have been characterized by high disease burden and sBCMA “sink effect,” T-cell exhaustion, and antigen escape (82). High disease burden and extramedullary disease are characterized by high levels of sBCMA, factors which have been implicated in resistance to BCMA- and GPRC5d-targeting bispecific antibodies (17, 33, 53, 83–86). High sBCMA levels attenuate BCMA-directed bispecific antibody binding and cytolytic activity in *in vitro* studies (87). Strategies aimed at mitigating high sBCMA levels and high disease burden as mechanisms of resistance include the development of bispecific antibodies that bind with higher affinity to full length BCMA rather than sBCMA, higher bispecific antibody dose concentrations, combining anti-BCMA bispecific with gamma-secretase inhibitors to increase the density of BCMA molecules on MM cells (and decrease sBCMA), and combining bispecific antibodies with other anti-myeloma agents to help debulk high burden disease (19, 82, 85, 86).

T-cell exhaustion has been shown to correlate with poor responses to bispecific antibodies as well as correlate with high-burden disease. Nonresponders/patients that exhibit primary refractoriness to bispecific antibodies have been shown to have lower peripheral CD8 T-cell counts, higher frequency of regulatory T cells, and higher overall frequencies of T cells that express markers associated with T-cell exhaustion such as PD-1, TIM-3, TOX, TCF-1, TIGIT, LAG-3 and CD38, in both bone marrow and peripheral blood at baseline (83, 88, 89). While data has shown that patients who respond to a BCMA-bispecific antibody and relapse will subsequently respond to a GPRC5d antibody suggesting that T-cell exhaustion may not pay as an important of a role in acquired resistance to bispecific antibodies, correlative studies from these patients have in fact shown evidence of T-cell exhaustion (89, 90). *In vitro* and *in vivo* work has shown that combining bispecific antibodies with immunomodulatory drugs such as pomalidomide can potentiate T-cell activation but exacerbates T-cell exhaustion while cyclophosphamide reduces tumor burden, depletes regulatory T cells and prevents T-cell exhaustion (91). Multiple studies are underway as aforementioned that combine bispecific antibodies with immunomodulatory drugs, cereblon modulators, anti-CD38 antibodies and PD-1 inhibitions and these therapeutic strategies may mitigate T-cell exhaustion and enhance the efficacy of bispecific antibodies in myeloma.

Antigen escape has been shown to be the predominant mechanism of acquired resistance to anti-BCMA and GPRC5d-targeting bispecific antibodies (92–94). BCMA antigenic loss, resulting from biallelic or monoallelic deletions and BCMA extracellular domain mutations have been observed in post-BCMA bispecific antibody relapses (92).

Mutations in the BCMA extracellular domain between amino acids in positions 27 (arginine) and 34 (proline) combined with monoallelic loss of TNFRSF17 (chr. 16p) has been observed in post-teclistamab and elranatamab relapses with these mutations abrogating the binding of these BCMA bispecific antibodies to BCMA (92). Trispecific antibodies which target two plasma cell antigens or combining a GPRC5d and BCMA-targeting bispecific antibody as in the RedirecTT-1 trial (NCT04586426), may help overcome antigen escape by eradicating clonal variants expressing low or no antigen (82).

## Discussion and future directions

The efficacy of bispecific antibodies in heavily pre-treated RRMM patient populations has led to promising new therapeutic options for patients with triple-class and penta-drug refractory myeloma who’ve historically had a poor prognosis of around 6 months. Pivotal data from the phase I/II studies of teclistamab, (MajesTEC-1), elranatamab (MagnetisMM-1), talquetamab (MonumenTAL-1) and cevostamab (NCT03275103) show ORRs of around 65% and a median PFS of around 11 months in RRMM patient populations (17, 33, 53, 65). Given the progressive T-cell dysfunction that is associated with disease progression and relapsed/refractory disease in patients with multiple myeloma, it is plausible that using bispecific T-cell engaging antibodies in earlier lines of therapy may produce deeper response rates and longer remissions (95). Several studies are underway evaluating bispecific antibodies in newly diagnosed MM vs. standard of care such as the MajesTEC-2,-4, -5, and -7 studies with teclistamab, the MagnetisMM-6 and-7 studies with elranatamab, the LINKER-MM4 study with linvoseltamab, and the GEM-TECTAL study with talquetamab. There are several studies evaluating bispecific antibodies in high-risk smoldering myeloma patients such as LINKER-SMM1 (NCT05955508) with linvoseltamab, Immuno-PRISM (NCT05469893) with teclistamab, and the REVIVE (NCT06100237) study of teclistamab or talquetamab in combination with daratumumab. The Immuno-PRISM study, a randomized phase II study of teclistamab vs. lenalidomide and dexamethasone in patients with high-risk smoldering myeloma has reported preliminary results. The primary objective of the study is the CR rate and 19 patients have been enrolled into the study and 12 have been treated with teclistamab to date. The CRS rate was 75% (all grade 1 except 2 patients with grade 2) and ≥grade 3 neutropenia and thrombocytopenia occurred in 4 patients and 1 patient, respectively. Infections occurred in 9 patients but only 1 patient had a grade 3 sinus infection. There were no reports of ICANS or delayed neurotoxicity. The ORR was 100% with 42% achieving a CR, 25% a VGPR, and 33% a PR. Of the 8 MRD evaluable patients treated with teclistamab, the MRD negative rate at 10^-6^ was 100%, Out of the 3 patients in the control arm of lenalidomide and dexamethasone, the ORR was 66% without any CR to date (96).

Multiple studies are underway evaluating the addition of other anti-myeloma agents to bispecific antibodies to enhance efficacy and overcome mechanisms of resistance as shown in **Tables 1**-**5**. In preclinical *in vivo* models of MM, the addition of lenalidomide and pomalidomide to AMG-701 (a BCMA x CD3 bispecific antibody), appeared to enhance the activity of AMG-701 as evidence by augmented T-cell–dependent cellular cytotoxicity against MM cell lines and autologous RRMM patient cells. The IMiDs lenalidomide and pomalidomide further upregulated AMG 701–induced patient T-cell differentiation toward memory phenotypes, associated with increased CD8/CD4 ratios, increased stem cell–like memory T cells and decreases in T regulatory cells which have previously been implicated in the immunosuppressive tumor microenvironment associated with resistance to bispecific antibodies in preclinical models of MM (91, 97). The addition of pomalidomide to talquetamab in preclinical models of MM has also shown to enhance the anti-MM activity of talquetamab and showed a trend towards a lower T regulatory cell frequency with combination treatment (52). Anti-CD38 monoclonal antibodies like daratumumab have immunomodulatory properties via the eradication of CD38-expressing regulatory T and B cells, and myeloid-derived suppressor cells which results in results in CD4+ and CD8+ T-cell expansion (67). Preclinical models of MM have shown that the addition of daratumumab to teclistamab and talquetamab enhances the anti-MM activity of these bispecific antibodies (52, 98).

Efforts to overcome mechanisms of resistance to bispecific antibodies are also underway. T-cell exhaustion and antigen escape are the two main purported mechanisms of resistance to bispecific antibodies. Combining bispecific antibodies with PD-1 inhibitors may prevent T-cell exhaustion and studies are underway combining talquetamab or teclistamab with a PD-1 inhibitor (TRIMM3 trial; NCT05338775). Additionally, bispecific antibodies targeting macrophages like ISB 1442 are in clinical development as a way to bypass T-cell exhaustion and NK-cell engaging bispecific antibodies in clinical development will also bypass T-cell exhaustion. Combining two bispecific antibodies like teclistamab and talquetamab as in the RedirecTT-1 study (NCT04586426) and use of trispecific antibodies targeting two plasma cell antigens can help prevent and overcome antigen escape.

Clinical trials evaluating BCMA, GPRC5d and FcRH5-targted bispecific antibodies have reported high infection rates ranging from 40-80% of patients on the trials. Chronic activation of T cells with bispecific antibody treatment may lead to T-cell exhaustion, which in turn reduces the ability of T cells to fight infection (99). Teclistamab was noted to induce rapid depletion of peripheral blood B cells and abolished normal plasma cells in ex vivo assays from 135 patients treated at the RP2D of teclistamab in the MajesTEC-1 study. Additionally, teclistamab reduced polyclonal immunoglobulin levels without recovery in patients who remained on teclistamab and responses to certain vaccines were severely impaired in patients treated with teclistamab. The use of intravenous immunoglobulin (IVIG) was associated with a lower risk of serious infections among patients treated with teclistamab at 6 months; 5.3% infections for patients on IVIG vs. 54.8% infection for patients not receiving IVIG (100). In a retrospective single center study of 37 patients treated with BCMA x CD3-targeting bispecific antibodies, 15 (41%) patients experienced a ≥grade 3 infection with most (84%) infections occurring during disease remissions. Among the 26 patients who responded to anti-BCMA bispecific antibody therapy, profound hypogammaglobulinemia occurred in 100% and receiving IVIG lowered the rate of a ≥grade 3 infection by 90% (101). Efforts and recommendations to mitigate infections with bispecific antibody treatment are ongoing (102).

With the advent of bispecific antibodies, CAR-T cell therapies, and antibody drug conjugates, many of which target the same malignant plasma cell antigens, data on how to best sequence these agents is starting to emerge. Patients who received teclistamab, elranatamab, talquetamab and cevostamab after prior BCMA-targeting therapies achieved ORRs of 40%, 54%, 50%, and 47%, respectively (103). However, patients who received ide-cel after prior anti-BCMA bispecific antibody exposure (n=7/50) achieved an ORR of 0% and a median PFS of 2.83 months and patients who received cilta-cel after prior anti-BCMA bispecific antibody exposure (n=7/20) achieved an ORR of 57.1% with median PFS of 5.3 months which is much lower than the ORRs and mPFS seen in BCMA-naïve patients and suggests that perhaps it is best to use anti-BCMA-CAR-T cell therapy first followed by anti-BCMA bispecific antibody when sequencing anti-BCMA directed therapies (104, 105). Teclistamab has shown efficacy in RRMM patients previously treated with prior BCMA-targeted therapy. In cohort C of the MajecTEC-1 trial, 38 patients had received a median of 6 prior lines of therapy including 16 patients (64%) who had received prior BCMA-targeting antibody drug conjugate and 11 (44%) patients who received prior BCMA-targeting CAR-T. In the BCMA-directed antibody drug conjugate-exposed and anti-BCMA CAR-T-exposed patients, the ORRs were 38% and 45%, respectively (28). In a pooled analysis of 86 patients from the MagnetisMM-1, -3, and -9 studies that evaluated patients treated with elranatamab who had received prior BCMA-directed therapy, 54.7% of patients were penta-drug refractory and patients had received a median of 7 prior lines of therapy, including BCMA-directed antibody drug conjugate (67.4%) and CAR T-cells (41.9%) with 9.3% having received both. Among patients who received antibody drug conjugate and CAR-T cells respectively, 79.3% and 27.8% were refractory to ADC and CAR-T cells. The ORR was 45.3% with ≥CR achieved in 17.4% of patients. The ORR for patients with prior BCMA-directed antibody-drug conjugate and CAR-T cells was 41.4% and 52.8%, respectively. Among responders, median time to objective response was 1.9 months. After a median follow-up of 10.3 months, the median PFS was 4.8 months and the median OS was not reached by 10 months, with a rate of 60.1% at 9 months (106). Talquetamab and cevostamab also show activity in patients who have received prior anti-BCMA bispecific antibodies with ORRs of 52% and 33.3%, respectively (55, 65). With the continued development of CAR-T cell therapies, bispecific antibodies, and antibody drug conjugates targeting a multitude of plasma cell antigens, sequencing data will be pivotal to maximize the ORR, PFS and OS of patients with RRMM.

**Figure 2:**
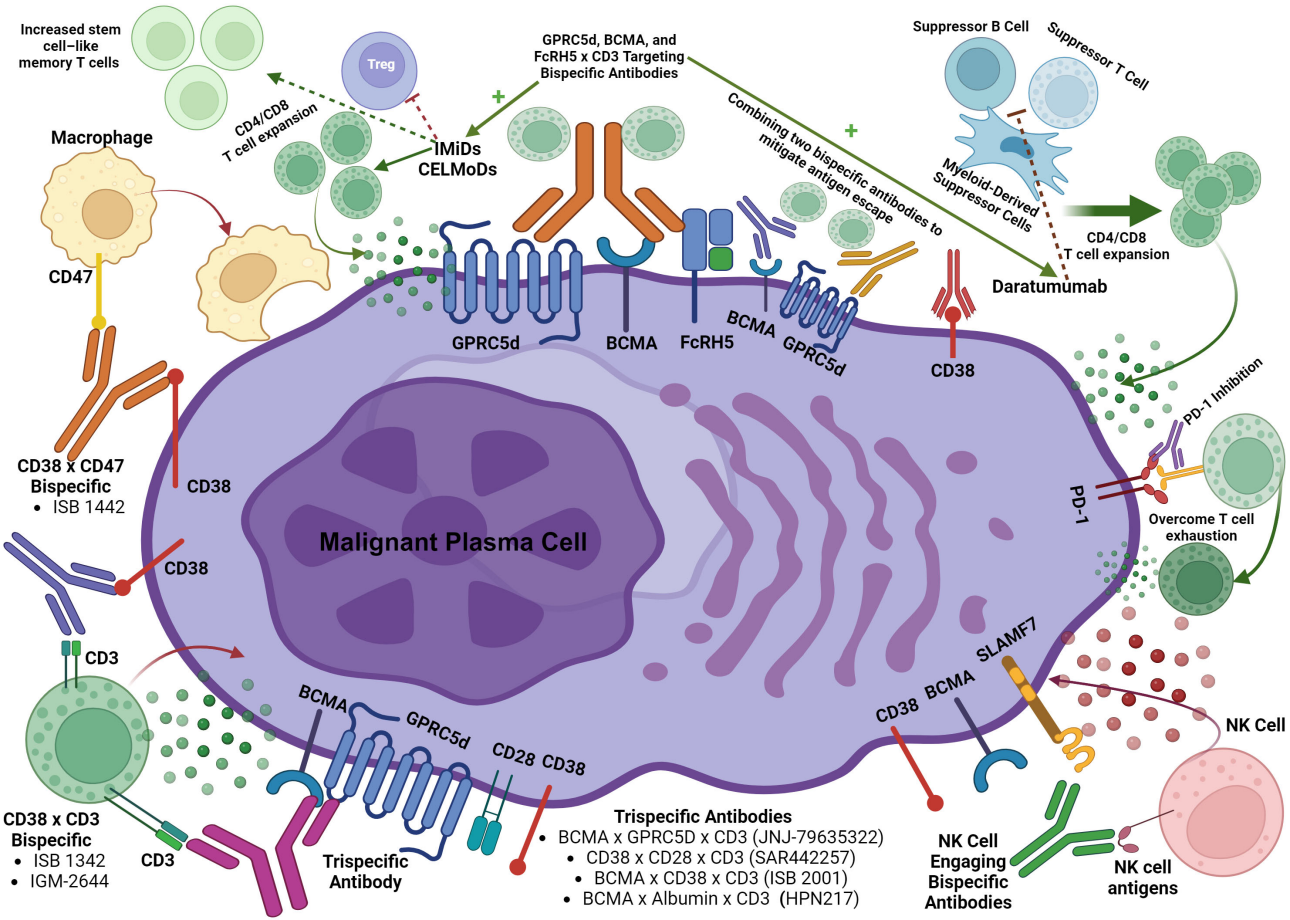
Future directions with bispecific antibodies in multiple myeloma.

Future directions for bispecific antibodies in multiple myeloma are illustrated in **Figure 2**.

## Conclusions

Bispecific antibodies targeting malignant plasma cell antigens and T-cell antigens are showing unprecedented response rates in RRMM, including in triple-class refractory and penta-drug refractory patients with well-defined toxicity profiles (**Table 7**). Bispecific antibodies targeting BCMA, GPRC5d and FcRH5 on myeloma cells and CD3 on T cells are the most advanced in clinical development and show efficacy in RRMM patient populations previously exposed to other T-cell directed therapies, even after sequentially targeting the same plasma cell antigen. Emerging data and ongoing clinical trials are demonstrating the potency and the potential of bispecific antibodies targeting CD3 and other plasma cell antigens such as CD38. Additionally, trispecific antibodies targeting two plasma cell antigens and CD3 on T cells are beginning to emerge as are bispecific and trispecific antibodies targeting malignant plasma cell antigens and NK-cell antigens. While the main side effects of these agents are CRS which can be managed, recurrent infections remain problematic as do some of the oral and skin/nail toxicities of GPRC5d-targeting bispecifics. Mechanisms of resistance to bispecific antibodies are well characterized and studies are underway to mitigate mechanisms of resistance by combining bispecifics with other anti-myeloma agents. While most RRMM patients treated with bispecific antibodies eventually relapse, clinical trial data is eagerly awaited evaluating bispecific antibodies in earlier lines of therapy and in smoldering myeloma to see if functional cures can be achieved.

**Table 7:** Summary of efficacy and safety of bispecific antibodies in relapsed refractory multiple myeloma.

Agent	BCMA-Directed BsAb						GPRC5d-Directed BsAb		FcRH5-Directed BsAb
	Teclistamab (17)	Elranatamab (34)	Linvoseltamab (37, 38)	ABBV-383 (41)	Alnuctamab (44)	WVT078 (45)	Talquetamab (53)^***^	Forimtamig (62)	Cevostamab (65)
Median Prior Lines	5	5	5	5	4	5	6	IV: 5 SQ: 4	6
Median Age, Y	64	68	66	68	64	64	64	IV: 62 SQ: 54	64
Triple-Class Refractory (%)	77.6	100	81	82	63	51.5	75	IV: 62.3 SQ: 73.1	85
Penta-Drug Refractory (%)	30.3	70.7	N/A	35	19	18.2	25	IV: 30.6 SQ: 42.3	N/A
ORR,%	63	61	71*	57	54	38.5**	405 μg QW: 70 800 μg Q2W: 64	IV: 71.4 SQ: 60.4	90mg: 36.7 160mg: 54.5
MRD- Rate (≥10^-5^), in patients who achieved ≥CR, %	46 (n=30)	89.7 (n=29)	N/A	81.2 (n=16)	100 (n=28)	66.6 (n=8)	69 (n=11)	N/A	N/A
DOR, months	18.4	NR (71.5% at 15mo)	NR* (79% at 12mo)	NR (79.9% at 12mo)	N/A	7.6	405 μg QW: 10.2 800 μg Q2W: 7.8	N/A	15.6
mPFS, months	11.3	NR (50.9% at 15mo)	NR*(66% at 12mo)	10.4	10.1	N/A	N/A	N/A	N/A
mOS, months	18.3	NR (56.7% at 15mo)	N/A	N/A	N/A	N/A	N/A	N/A	N/A
CRS, ≥grade 3 (%)	76.4, 44.8	57.7, 0	45, 1*	57, 2	56, 0	60.1, 12.1	405 μg QW: 77, 3 800 μg Q2W: 80, 0	IV: 82.4, 2 SQ: 77.8, 1.9	80, 1.3
ICANS, ≥grade 3 (%)	3, 0	3.4, 0	N/A	1.6, 0	2.7, 0	0	405 μg QW: 10, 0 800 μg Q2W: 5, 0	8.6, 1.9	0.3/3.6 step-up: 4.5, 0 3.6/target step-up: 21.2, 0
Infections, ≥grade 3 (%)	76.4, 44.8	69.9, 39.8	59.8, 36.8	41, 22.5	62, 16	58, 12	405 μg QW: 47, 7 800 μg Q2W: 34, 7	IV: 56.9, 19.6 SQ: 37, 24.1	42.5, 18.8

*at RP2D.

** ≥48 µg/kg.

***SQ dosing only.

BsAb, bispecific antibody CRS, cytokine release syndrome; mDOR, median duration of response ICANS, immune effector cell-associated neurotoxicity syndrome; MRD-, minimal residual disease negativity; NR, not reached; N/A, not reported; ORR, overall response rate; mPFS, median progression free survival; mOS, median overall survival; RP2D recommended phase 2 dose.

## Author contributions

RP: Conceptualization, Writing – original draft, Writing – review & editing. SA: Supervision, Writing – original draft, Writing – review & editing. CC: Supervision, Writing – original draft, Writing – review & editing.
